# Falls and Fall Injuries Among Adults with Arthritis — United States, 2012

**Published:** 2014-05-02

**Authors:** Kamil E. Barbour, Judy A. Stevens, Charles G. Helmick, Yao-Hua Luo, Louise B. Murphy, Jennifer M. Hootman, Kristina Theis, Lynda A. Anderson, Nancy A. Baker, David E. Sugerman

**Affiliations:** 1 Division of Population Health, National Center for Chronic Disease Prevention and Health Promotion, CDC; 2 Division of Unintentional Injury Prevention; National Center for Injury Prevention and Control, CDC; 3 Department of Occupational Therapy, University of Pittsburgh

Falls are the leading cause of injury-related morbidity and mortality among older adults, with more than one in three older adults falling each year,* resulting in direct medical costs of nearly $30 billion (1). Some of the major consequences of falls among older adults are hip fractures, brain injuries, decline in functional abilities, and reductions in social and physical activities (2). Although the burden of falls among older adults is well-documented (1,2), research suggests that falls and fall injuries are also common among middle-aged adults (3). One risk factor for falling is poor neuromuscular function (i.e., gait speed and balance), which is common among persons with arthritis (2). In the United States, the prevalence of arthritis is highest among middle-aged adults (aged 45–64 years) (30.2%) and older adults (aged ≥65 years) (49.7%), and these populations account for 52% of U.S. adults (4). Moreover, arthritis is the most common cause of disability (5). To examine the prevalence of falls among middle-aged and older adults with arthritis in different states/territories, CDC analyzed data from the 2012 Behavioral Risk Factor Surveillance System (BRFSS) to assess the state-specific prevalence of having fallen and having experienced a fall injury in the past 12 months among adults aged ≥45 years with and without doctor-diagnosed arthritis. This report summarizes the results of that analysis, which found that for all 50 states and the District of Columbia (DC), the prevalence of any fall (one or more), two or more falls, and fall injuries in the past 12 months was significantly higher among adults with arthritis compared with those without arthritis. The prevalence of falls and fall injuries is high among adults with arthritis but can be addressed through greater dissemination of arthritis management and fall prevention programs in clinical and community practice.

BRFSS is an annual, random-digit–dialed landline and cellphone survey representative of the noninstitutionalized adult population aged ≥18 years of the 50 states, DC, and the U.S. territories. In 2012, a total of 338,734 interviews with persons aged ≥45 years were completed, and data from 50 states, DC, Puerto Rico, and Guam are included in this report (the U.S. Virgin Islands did not collect BRFSS data). Response rates ranged from 27.7% to 60.4%, with a median of 45.2%.†

Respondents were defined as having arthritis if they answered “yes” to the question, “Have you ever been told by a doctor or other health professional that you have some form of arthritis, rheumatoid arthritis, gout, lupus, or fibromyalgia?” The BRFSS survey asks about falls in the past year, explaining to the respondent that, “By a fall, we mean when a person unintentionally comes to rest on the ground or another lower level.” Respondents were considered to have fallen if they answered the question, “In the past 12 months, how many times have you fallen?” with a number of one or more. The number of falls was analyzed as a categorical variable (zero, one, or two or more) and as a dichotomous variable (yes or no). Those who reported one or more falls were also asked, “How many of these falls caused an injury? By an injury, we mean the fall caused you to limit your regular activities for at least a day or to go see a doctor?” Injury from any fall was categorized as a dichotomous variable (yes or no).

All analyses used sampling weights to account for the complex sample design, nonresponse, noncoverage, and cellphone-only households. Since 2011, iterative proportional weighting (raking) has been used and shown to reduce nonresponse bias and error within estimates compared with post-stratification weighting.§ Thus, 2012 estimates should not be compared with estimates made before 2011. The unadjusted prevalence of any fall (one or more in the past 12 months) with 95% confidence intervals (CIs) for combined state/territory data was used to assess the similarity of prevalence for two age groups (45–64 and ≥65 years). State-specific unadjusted prevalence of fall outcomes among adults aged ≥45 years with and without arthritis are available at http://www.cdc.gov/arthritis/data_statistics/prevalence-injuries-falls-by-state.htm. Age-adjusted estimates were standardized to the year 2000 U.S. standard population using five age-groups (45–54, 55–64, 65–74, 75–84, and ≥85 years). Age-adjusted estimates were presented and used to compare the prevalence of one fall, any fall, two or more falls, and fall injuries by arthritis status across states/territories. In addition, medians and ranges for all states and DC were determined for each fall outcome. For all comparisons, differences were considered statistically significant if the CIs of the age-adjusted estimates did not overlap.

The unadjusted prevalence of having experienced any fall in the past 12 months was similar for adults aged 45–64 years (25.5%) and ≥65 years (27.0%); therefore, state-specific findings for the combined ≥45 years age group are reported. Overall the unadjusted median state prevalence of arthritis among adults aged ≥45 years was 40.1% (range = 31.0%–51.9%), and the median prevalence of one fall, two or more falls, and fall injuries in the preceding year was 13.8% (range = 8.8%–16.7%), 13.3% (range = 6.1%–21.0%), and 9.9% (range = 4.5%–13.3%), respectively.

In analyses of adults with arthritis, the age-adjusted median prevalence for one fall was 15.5% (range = 10.7% in Wisconsin to 20.1% in Washington), for two or more falls was 21.3% (range = 7.7% in Wisconsin to 30.6% in Alaska), and for fall injuries was 16.2% (range = 8.5% in Wisconsin to 22.1% in Oklahoma) (Table). Among adults without arthritis, the age-adjusted median prevalence of one fall, two or more falls, and fall injuries was 12.1% (range = 7.7% in Wisconsin to 15.1% in Wyoming), 9.0% (range = 4.1% in Wisconsin to 14.6% in Alaska), and 6.5% (range = 2.7% in Wisconsin to 9.0% in Alaska), respectively. Within every state and territory except Guam, the prevalence of two or more falls and fall injuries was significantly higher for those with arthritis compared with those without arthritis (Table). The age-adjusted median prevalence of one fall, any fall, two or more falls, and fall injuries was 28%, 79%, 137%, and 149% higher (relative differences), respectively, among adults with arthritis compared with adults without arthritis.

In 2012, 46 states and DC had an age-adjusted prevalence of any fall in the past 12 months of ≥30% among adults with arthritis, and 16 states had an age-adjusted prevalence of any fall of ≥40% (Figure). Among adults without arthritis, no state/territory had an age-adjusted prevalence of falls ≥30% or had a significantly higher age-adjusted prevalence of falls compared with adults with arthritis.

## Discussion

In all 50 states and DC, the prevalence of any fall (one or more), two or more falls, and fall injuries in the past 12 months was significantly higher among adults aged ≥45 years with arthritis compared with those without arthritis. Among persons with arthritis, about half of all states had a prevalence of multiple falls (two or more) ranging from 21% to 31% and a prevalence of fall injuries ranging from 16% to 22%. In 45 states and DC, the age-adjusted prevalence of any fall among adults with arthritis was ≥30%; in contrast, the prevalence of any fall in adults without arthritis did not reach 30% in any state. Finally, the age-adjusted median prevalence of two or more falls and fall injuries among adults with arthritis was approximately 2.4 and 2.5 times higher, respectively, than those without arthritis.

The 2010 U.S. Census reported 81.5 million adults (26.4% of the population) aged 45–64 and 40.3 million persons (13.0%) aged ≥65 years. The projected rapid growth in the population aged ≥65 years¶ and the increase in adults with arthritis (an estimated 67 million by 2030) (6) demonstrate the need for increasing fall prevention efforts.

Public health approaches to prevent falls among older adults have focused on modifying fall risk factors (e.g., muscle weakness in the legs, gait and balance problems, psychoactive medication use, poor vision, and environmental hazards such as slippery surfaces or tripping hazards), in addition to identifying and treating the symptoms of chronic conditions that increase fall risk, such as arthritis.** Public health approaches to preventing poor outcomes among adults with arthritis have focused on evidence-based self-management education and physical activity interventions†† that have been proven to reduce pain and improve function by correcting muscle weakness and balance dysfunction. Combining arthritis exercise programs with proven fall prevention intervention might reduce the risk for falls in this at-risk population.

Effective fall prevention interventions can be multifaceted, but the most effective single strategy involves exercise or physical therapy to improve gait, balance, and lower body strength, which have been shown to reduce fall risk by 14%–37% (7). For an exercise program to be effective in reducing falls it must 1) focus on improving balance, 2) become progressively more challenging, and 3) involve at least 50 hours of practice (e.g., a 1-hour Tai Chi class taken twice a week for 25 weeks) (8). As a form of exercise, Tai Chi is an effective fall prevention intervention§§ that has also been shown to improve neuromuscular function (9). However, the effects of Tai Chi intervention programs on arthritis-specific outcomes are still being evaluated; therefore, Tai Chi is not currently endorsed for use by the 12 CDC-funded state arthritis programs that disseminate arthritis-appropriate, evidence-based intervention programs for use in local communities. Existing arthritis physical activity interventions, especially EnhanceFitness and Fit and Strong¶¶ might reduce the risk for falls and fall injuries but have not yet been evaluated for these outcomes.

The findings in this report are subject to at least four limitations. First, data in BRFSS are based on self-report; therefore, arthritis status, falls, and a fall injury might be misclassified. The case-finding question used in BRFSS to assess arthritis status has been judged to be sufficiently sensitive and specific for public health surveillance purposes among those aged ≥65 years, but it is less sensitive for those aged <65 years than is desirable (10); however, recall bias might contribute to an underestimate of self-reported falls. Conversely, the broad definition of a fall injury might have led participants to report minor falls as injurious, resulting in an overestimate. Second, because BRFSS is a cross-sectional survey, the temporal sequence of arthritis and falls could not be established. Nonetheless, a meta-analysis of seven longitudinal studies showed that persons with arthritis have more than a two-fold increased risk for falls (2). Third, no BRFSS questions assess the severity, location, or type of arthritis, which might affect falls and fall injuries differently. Finally, the 2012 median survey response rate for all states and DC was 45.2% and ranged from 27.7% to 60.4%; lower response rates can result in nonresponse bias, although the application of sampling weights is expected to reduce nonresponse bias.

What is already known on this topic?

In the United States, arthritis, falls, and fall injuries are highly prevalent conditions among middle-aged (aged 45–64 years) and older (aged ≥65 years) adults. Falls are the leading cause of injury-related morbidity and mortality among older adults; meanwhile, arthritis remains the most common cause of disability.

What is added by this report?

During 2012, for all 50 states and the District of Columbia, the prevalence of any fall (one or more), two or more falls, and fall injuries in the past 12 months was significantly higher among adults with arthritis compared with those without arthritis. Moreover, among adults with arthritis, the age-adjusted median prevalences of one fall, any fall, two or more falls, and fall injuries were 28%, 79%, 137%, and 149% higher, respectively, compared with adults without arthritis.

What are the implications for public health practice?

The burden of falls and fall injuries is high among adults with arthritis but can be addressed through greater dissemination of arthritis management and fall prevention programs in clinical and community practice.

The number of adults with arthritis is expected to increase steadily through at least 2030 (6), putting more adults at higher risk for falls and fall injuries. Efforts to address this growing public health problem require raising awareness about the link between arthritis and falls, evaluating evidence-based arthritis interventions for their effects on falls, and implementing fall prevention programs more widely through changes in clinical and community practice.

## Figures and Tables

**FIGURE:**
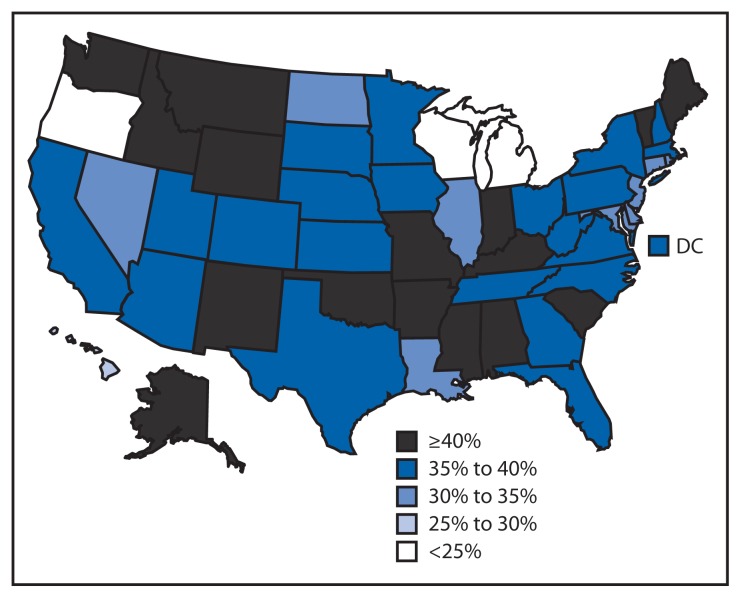
Age-standardized prevalence of having one or more falls in the past 12 months among adults aged ≥45 years with arthritis — Behavioral Risk Factor Surveillance System, United States, 2012

**TABLE:** Weighted age-adjusted prevalence of falls* and fall injuries in the past 12 months,† among adults aged ≥45 years with and without arthritis,§ by state/territory¶ — Behavioral Risk Factor Surveillance System, United States, 2012

State/Area	One fall						Two or more falls						Fall injury					
		
	Sample size**	Population**	Arthritis		No arthritis		Sample size**	Population**	Arthritis		No arthritis		Sample size**	Population**	Arthritis		No arthritis	
					
			%	(95% CI)	%	(95% CI)			%	(95% CI)	%	(95% CI)			%	(95% CI)	%	(95% CI)
Alabama	980	256,858	16.0	(14.2–18.0)	11.3	(10.0–12.9)	1,101	324,718	26.0	(23.7–28.4)	9.3	(7.9–11.0)	835	228,719	18.7	(16.6–20.9)	5.4	(4.5–6.4)
Alaska	409	36,579	15.0	(12.3–18.1)	14.3	(11.9–17.0)	534	53,317	30.6	(26.5–35.0)	14.6	(12.4–17.1)	350	35,369	20.8	(17.4–24.7)	9.0	(7.1–11.5)
Arizona	813	328,358	16.3	(13.3–19.7)	12.1	(10.4–14.0)	732	299,524	21.9	(18.6–25.7)	8.2	(6.8–9.9)	606	262,168	18.1	(15.1–21.5)	7.0	(5.8–8.5)
Arkansas	548	146,006	14.4	(12.3–16.7)	11.0	(9.4–12.8)	678	209,133	27.8	(24.8–30.9)	11.5	(9.8–13.5)	488	144,016	20.9	(18.2–23.8)	6.0	(4.9–7.4)
California	1,309	1,712,404	15.6	(13.9–17.5)	13.3	(12.0–14.8)	1,182	1,563,446	19.4	(17.4–21.6)	9.7	(8.6–10.8)	1,027	1,334,678	15.6	(13.9–17.5)	6.5	(5.6–7.4)
Colorado	1,287	288,047	18.2	(16.4–20.2)	14.2	(13.0–15.5)	1,122	243,734	19.8	(17.8–22.0)	9.5	(8.5–10.6)	909	211,557	17.8	(15.9–19.9)	7.1	(6.2–8.0)
Connecticut	845	186,356	14.0	(12.1–16.1)	11.1	(9.8–12.5)	732	177,566	19.9	(17.3–22.8)	7.9	(6.8–9.0)	641	148,629	17.1	(14.8–19.8)	6.0	(5.1–7.1)
Delaware	473	46,888	15.4	(12.7–18.5)	10.3	(8.8–12.1)	422	44,498	19.4	(16.4–22.7)	7.2	(5.9–8.8)	365	35,880	14.0	(11.5–16.8)	6.6	(5.4–8.0)
DC	396	31,436	13.9	(10.6–17.9)	15.1	(12.4–18.2)	315	27,168	24.2	(19.3–29.8)	7.5	(6.0–9.3)	291	26,465	20.0	(15.6–25.1)	8.0	(5.9–10.7)
Florida	749	968,371	14.3	(11.8–17.2)	10.4	(9.0–12.1)	721	971,220	20.8	(18.0–23.9)	7.2	(6.1–8.6)	669	862,502	17.4	(14.7–20.4)	6.3	(5.2–7.5)
Georgia	597	479,332	16.6	(14.0–19.5)	12.0	(10.4–13.8)	602	476,094	22.4	(19.6–25.5)	8.4	(7.1–10.0)	511	390,040	18.4	(15.9–21.3)	6.5	(5.4–7.9)
Hawaii	613	67,584	15.2	(12.4–18.6)	10.4	(8.9–12.2)	451	45,385	13.5	(11.0–16.5)	6.2	(5.0–7.6)	418	41,177	13.3	(10.6–16.7)	5.0	(4.2–6.0)
Idaho	707	86,883	15.0	(12.4–18.0)	14.4	(12.3–16.9)	761	93,282	25.2	(21.3–29.5)	11.2	(9.5–13.2)	570	67,320	18.9	(15.6–22.8)	7.6	(6.2–9.3)
Illinois	593	678,156	15.5	(13.3–18.0)	12.3	(10.6–14.2)	476	567,290	16.6	(14.0–19.4)	8.0	(6.6–9.7)	408	464,542	15.2	(12.8–18.0)	5.1	(4.1–6.3)
Indiana	888	374,522	16.9	(15.0–18.9)	13.7	(12.3–15.3)	926	381,394	23.8	(21.6–26.2)	10.0	(8.7–11.4)	663	275,651	16.8	(14.9–18.9)	6.9	(5.9–8.1)
Iowa	789	186,009	15.2	(13.3–17.4)	15.0	(13.5–16.5)	674	175,584	22.8	(20.2–25.5)	9.9	(8.7–11.4)	500	125,108	15.9	(13.7–18.3)	6.7	(5.7–7.8)
Kansas	1,295	159,978	16.5	(14.9–18.3)	12.9	(11.8–14.0)	1,205	156,339	22.4	(20.3–24.6)	9.8	(8.8–10.9)	824	103,103	15.3	(13.5–17.2)	5.8	(5.1–6.7)
Kentucky	1,144	229,858	15.4	(13.8–17.2)	11.7	(10.3–13.2)	1,319	298,532	26.0	(23.6–28.6)	10.3	(8.9–11.9)	1,008	213,288	18.4	(16.5–20.6)	6.2	(5.2–7.4)
Louisiana	769	181,584	12.2	(10.4–14.4)	9.1	(7.9–10.5)	910	222,659	21.3	(18.7–24.2)	6.7	(5.5–8.1)	607	151,012	12.4	(10.6–14.6)	5.9	(4.9–7.2)
Maine	1,138	92,883	16.8	(15.1–18.6)	13.8	(12.6–15.1)	1,136	96,548	24.3	(22.2–26.6)	10.7	(9.6–11.8)	840	69,631	18.4	(16.5–20.4)	6.8	(5.9–7.7)
Maryland	1,217	278,273	15.6	(13.5–18.0)	10.9	(9.7–12.1)	991	219,260	15.1	(13.3–17.0)	6.7	(5.8–7.8)	864	187,961	12.9	(11.3–14.8)	5.6	(4.8–6.6)
Massachusetts	2,079	352,749	16.4	(14.7–18.2)	11.8	(10.8–12.8)	1,762	293,545	18.6	(16.8–20.5)	7.6	(6.8–8.4)	1,653	267,905	16.2	(14.6–18.0)	6.4	(5.7–7.1)
Michigan	815	407,924	12.2	(10.8–13.9)	8.1	(7.1–9.2)	514	305,661	12.0	(10.2–14.1)	4.3	(3.6–5.3)	472	249,957	10.1	(8.5–12.0)	3.0	(2.4–3.8)
Minnesota	1,218	291,368	16.4	(14.5–18.6)	12.8	(11.7–13.9)	985	254,660	21.1	(18.6–23.7)	8.2	(7.3–9.2)	802	194,999	16.2	(14.1–18.7)	5.7	(5.0–6.5)
Mississippi	787	139,653	15.4	(13.5–17.5)	10.0	(8.7–11.5)	889	179,522	24.9	(22.5–27.5)	9.2	(7.8–10.7)	680	124,024	17.1	(15.1–19.3)	5.6	(4.7–6.8)
Missouri	764	360,504	18.1	(15.9–20.6)	12.6	(11.0–14.4)	756	379,648	24.1	(21.4–27.1)	10.0	(8.4–11.8)	605	284,659	18.6	(16.2–21.3)	6.9	(5.7–8.3)
Montana	922	63,860	16.8	(14.8–19.1)	13.3	(11.9–14.8)	1,111	78,636	25.5	(23.1–28.1)	14.0	(12.5–15.5)	742	49,480	17.0	(14.9–19.2)	7.9	(6.8–9.1)
Nebraska	2,218	114,065	18.5	(16.8–20.3)	14.5	(13.5–15.6)	1,886	91,793	19.0	(17.2–21.0)	9.4	(8.5–10.3)	1,445	70,856	15.8	(14.2–17.5)	6.5	(5.8–7.2)
Nevada	451	123,607	14.5	(11.5–18.2)	11.1	(9.2–13.4)	451	117,912	20.0	(16.5–23.9)	7.9	(6.5–9.6)	351	91,292	13.9	(11.1–17.2)	6.5	(5.0–8.3)
New Hampshire	853	81,481	16.3	(14.4–18.5)	12.9	(11.5–14.5)	859	83,990	19.8	(17.5–22.3)	11.0	(9.7–12.5)	661	63,234	15.5	(13.5–17.6)	7.8	(6.7–9.1)
New Jersey	1,273	392,045	14.2	(12.6–16.0)	9.9	(8.8–11.0)	974	311,829	15.8	(14.1–17.8)	5.9	(5.1–6.8)	964	295,364	14.1	(12.4–16.0)	5.5	(4.8–6.4)
New Mexico	871	115,409	16.5	(14.5–18.7)	13.4	(12.0–14.8)	912	123,436	26.0	(23.4–28.7)	11.0	(9.8–12.3)	743	98,863	19.6	(17.5–21.9)	7.7	(6.7–8.8)
New York	609	1,160,253	17.7	(14.9–20.9)	13.8	(11.9–15.9)	489	972,909	20.2	(16.9–23.8)	8.7	(7.2–10.5)	460	829,218	15.3	(12.9–18.2)	7.8	(6.4–9.5)
North Carolina	1,102	502,240	14.8	(13.1–16.6)	12.5	(11.2–13.8)	1,100	513,843	21.9	(19.9–24.1)	8.8	(7.8–10.1)	822	358,263	14.8	(13.1–16.6)	6.1	(5.3–6.9)
North Dakota	517	40,120	16.4	(13.8–19.4)	12.5	(10.9–14.4)	447	36,715	18.3	(15.3–21.7)	10.6	(8.9–12.6)	348	27,347	15.7	(12.8–19.1)	6.6	(5.4–8.2)
Ohio	1,242	619,185	14.8	(13.3–16.4)	11.8	(10.6–13.1)	1,300	616,621	20.8	(18.9–22.7)	8.4	(7.4–9.5)	1,034	492,055	16.1	(14.5–17.8)	6.3	(5.5–7.4)
Oklahoma	801	202,036	15.5	(13.7–17.5)	12.0	(10.7–13.5)	1,031	266,556	29.7	(27.1–32.4)	10.6	(9.3–12.0)	742	186,433	22.1	(19.8–24.6)	5.8	(4.9–6.9)
Oregon	427	170,229	13.8	(11.4–16.8)	8.6	(7.3–10.1)	280	109,037	10.6	(8.5–13.1)	4.9	(3.9–6.2)	263	100,791	9.4	(7.5–11.7)	4.1	(3.2–5.2)
Pennsylvania	2,056	775,966	16.9	(15.4–18.5)	12.8	(11.6–14.0)	1,838	651,072	19.2	(17.6–20.9)	7.6	(6.8–8.5)	1,534	538,263	14.6	(13.3–16.1)	6.6	(5.8–7.5)
Rhode Island	502	52,092	15.3	(13.0–17.8)	10.1	(8.6–11.7)	461	50,039	17.5	(15.0–20.3)	8.1	(6.7–9.8)	420	43,397	14.9	(12.7–17.4)	6.5	(5.4–7.7)
South Carolina	1,238	244,630	16.2	(14.3–18.2)	11.3	(10.1–12.7)	1,258	263,224	24.1	(21.9–26.5)	8.1	(7.1–9.3)	1,011	207,080	18.8	(16.8–21.0)	6.1	(5.2–7.2)
South Dakota	900	54,348	19.6	(16.4–23.2)	14.7	(12.7–17.0)	751	40,861	20.3	(17.2–23.8)	9.0	(7.5–10.8)	617	34,616	18.9	(15.7–22.5)	7.0	(5.7–8.7)
Tennessee	605	305,920	14.2	(12.2–16.5)	11	(9.4–12.7)	749	372,174	23.7	(21.3–26.3)	8.1	(6.8–9.6)	439	225,958	12.5	(10.6–14.6)	5.9	(4.8–7.2)
Texas	844	1,106,235	14.3	(12.3–16.7)	11.9	(10.4–13.6)	834	1,196,235	21.9	(19.3–24.8)	9.0	(7.8–10.3)	679	904,705	16.8	(14.4–19.5)	6.6	(5.6–7.7)
Utah	1,126	116,915	17.9	(16.0–20.0)	12.9	(11.7–14.2)	1,038	106,471	19.2	(17.3–21.3)	10.0	(8.9–11.2)	759	78,484	15.3	(13.5–17.2)	6.5	(5.7–7.5)
Vermont	691	42,124	15.7	(13.6–18.1)	14.4	(12.7–16.2)	766	48,216	26.3	(23.5–29.3)	12.4	(10.9–14.1)	514	30,740	17.1	(14.8–19.8)	7.2	(6.1–8.5)
Virginia	642	370,673	14.8	(12.8–17.0)	10.1	(8.8–11.5)	598	390,276	21.2	(18.5–24.1)	7.6	(6.5–8.8)	436	273,548	14.1	(12.0–16.3)	5.2	(4.3–6.2)
Washington	1,922	449,370	20.1	(18.3–22.0)	15.0	(14.0–16.1)	1,704	412,140	22.0	(20.3–24.0)	11.9	(10.9–13.0)	1346	326,695	18.4	(16.7–20.2)	8.5	(7.6–9.4)
West Virginia	479	97,758	12.9	(11.2–14.7)	10.3	(8.8–11.9)	598	131,714	23.3	(20.8–25.9)	9.8	(8.3–11.6)	380	79,390	13.8	(11.9–16.0)	5.5	(4.4–6.8)
Wisconsin	333	197,943	10.7	(8.5–13.5)	7.7	(6.2–9.6)	235	138,625	10.0	(7.7–12.8)	4.1	(3.1–5.5)	182	109,173	8.5	(6.3–11.5)	2.7	(1.9–3.9)
Wyoming	744	33,459	16.6	(13.8–19.7)	15.1	(13.2–17.3)	807	38,643	29.5	(25.8–33.6)	11.5	(9.8–13.5)	559	27,191	20.2	(17.0–23.8)	7.5	(6.2–9.1)
*Median* ††			*15.5*		*12.1*				*21.3*		*9.0*				*16.2*		*6.5*	
*Range* ††			*10.7–20.1*		*7.7–15.1*				*10.0–30.6*		*4.1–14.6*				*8.5–22.1*		*2.7–9.0*	
Puerto Rico	504	160,786	12.6	(10.9–14.6)	10.2	(8.8–11.7)	459	175,156	16.9	(14.5–19.5)	7.4	(5.3–10.3)	463	170,429	16.6	(14.4–19.2)	8.9	(7.5–10.6)
Guam	107	5,278	16.3	(11.5–22.6)	12.1	(8.7–16.6)	98	4,703	18.6	(12.3–27.0)	9.8	(8.3–11.7)	81	3,790	15.7	(9.9–23.9)	6.6	(4.4–9.9)

**Abbreviations:** CI = confidence interval; DC = District of Columbia.

* Falls were defined as self-reported number of falls in past 12 months.

† Injury from a fall was defined as self-reported injury caused by a fall in past 12 months that caused respondent to limit their regular activities for ≥1 days or to go see a doctor.

§ Doctor-diagnosed arthritis was defined based on a “yes” response to the question, “Have you ever been told by a doctor or other health professional that you have some form of arthritis, rheumatoid arthritis, gout, lupus, or fibromyalgia?”

¶ Includes all 50 states, DC, Puerto Rico, and Guam.

** Sample size represents the actual number with the outcome, whereas population is the weighted number of adults with the outcome.

†† Does not include Puerto Rico or Guam.

## Footnotes

* Information available at http://www.cdc.gov/injury/wisqars.

† The response rate was the number of respondents who completed the survey as a proportion of all eligible and likely eligible persons. Response rates for BRFSS were calculated using standards set by the American Association of Public Opinion Research response rate formula no. 4. Additional information available at http://www.cdc.gov/brfss/annual_data/2012/pdf/summarydataqualityreport2012_20130712.pdf.

§ Additional information available at http://www.cdc.gov/brfss/annual_data/2012/pdf/overview_2012.pdf.

¶ Additional information available at https://www.census.gov/prod/2010pubs/p25-1138.pdf.

** Additional information available at http://www.americangeriatrics.org/health_care_professionals/clinical_practice/clinical_guidelines_recommendations/2010.

†† Additional information available at http://www.cdc.gov/arthritis/interventions/marketing-support/compendium/docs/pdf/compendium-2012.pdf.

§§ Additional information available at http://www.cdc.gov/homeandrecreationalsafety/falls/preventfalls.html#compendium.

¶¶ Additional information available at http://www.cdc.gov/arthritis/interventions/marketing-support/compendium/docs/pdf/compendium-2012.pdf.

## References

[1] Stevens JA, Corso PS, Finkelstein EA, Miller TR (2006). The costs of fatal and non-fatal falls among older adults. Inj Prev.

[2] Rubenstein LZ, Josephson KR (2006). Falls and their prevention in elderly people: what does the evidence show?. Med Clin North Am.

[3] Talbot LA, Musiol RJ, Witham EK, Metter EJ (2005). Falls in young, middle-aged and older community dwelling adults: perceived cause, environmental factors and injury. BMC Public Health.

[4] CDC (2013). Prevalence of doctor-diagnosed arthritis and arthritis-attributable activity limitation—United States, 2010–2012. MMWR.

[5] CDC (2009). Prevalence and most common causes of disability among adults—United States, 2005. MMWR.

[6] Hootman JM, Helmick CG (2006). Projections of US prevalence of arthritis and associated activity limitations. Arthritis Rheum.

[7] Gillespie LD, Robertson MC, Gillespie WJ (2009). Interventions for preventing falls in older people living in the community. Cochrane Database Syst Rev.

[8] Sherrington C, Tiedemann A, Fairhall N, Close JC, Lord SR (2011). Exercise to prevent falls in older adults: an updated meta-analysis and best practice recommendations. N S W Public Health Bull.

[9] Jahnke R, Larkey L, Rogers C, Etnier J, Lin F (2010). A comprehensive review of health benefits of qigong and tai chi. Am J Health Promot.

[10] Sacks JJ, Harrold LR, Helmick CG, Gurwitz JH, Emani S, Yood RA (2005). Validation of a surveillance case definition for arthritis. J Rheumatol.

